# Administration of RAS Inhibitor before the Onset of Diabetic Nephropathy Counteracts the Adverse Effect of Chronic Hyperglycemia and Reduces the Augmentation of Urinary Albumin Excretion: A Retrospective Clinical Study

**DOI:** 10.1155/2018/9435401

**Published:** 2018-11-07

**Authors:** Hidenori Hirukawa, Shinji Kamei, Tomohiko Kimura, Atsushi Obata, Kenji Kohara, Fuminori Tatsumi, Masashi Shimoda, Shuhei Nakanishi, Tomoatsu Mune, Kohei Kaku, Hideaki Kaneto

**Affiliations:** Department of Diabetes, Endocrinology and Metabolism, Kawasaki Medical School, Japan

## Abstract

It is very important to explore how we can reduce urinary albumin excretion which is an independent risk factor for ischemic heart disease. In this study, we retrospectively evaluated the effects of RAS inhibitor therapy on diabetic nephropathy in Japanese subjects whose urinary albumin levels were within normal range. We enrolled 100 subjects with type 2 diabetes who did not take any renin-angiotensin system (RAS) inhibitor. We defined the subjects taking RAS inhibitor for more than 3 years as RAS inhibitor group. RAS inhibitor exerted protective effect on the progression of urinary albumin excretion in subjects with type 2 diabetes without diabetic nephropathy. In addition, RAS inhibitor exerted more protective effects on renal function especially in subjects with poor glycemic control. In conclusion, RAS inhibitor could protect renal function against the deleterious effect of chronic hyperglycemia in Japanese subjects with type 2 diabetes even before the onset of diabetic nephropathy.

## 1. Introduction

It is known that the augmentation of urinary albumin excretion (UAE) is associated with vascular endothelium disorder and is an independent risk factor for ischemic heart disease [1–5]. In addition, the increase of UAE leads to the ratio of cardiovascular disease-related mortality even before the onset of diabetic nephropathy [6, 7]. Therefore, it is very important to explore how we can reduce UAE. Hyperglycemia and subsequent activation of renin-angiotensin system (RAS) are associated with the development of diabetic nephropathy [8–12]. It was reported that RAS inhibitors suppressed the progression of overt diabetic nephropathy [13–16]. It remained unclear, however, whether RAS inhibitors exert such beneficial effects even before the onset of diabetic nephropathy. In this study, we retrospectively evaluated the effects of RAS inhibitor therapy on diabetic nephropathy in Japanese subjects whose urinary albumin levels were within normal range.

## 2. Methods

We enrolled the subjects with type 2 diabetes who visited Department of Diabetes, Endocrinology and Metabolism in Kawasaki Medical School and did not take any RAS inhibitor. We defined the subjects who started taking RAS inhibitor and continued for more than 3 years as RAS inhibitor group and the subjects who did not start any RAS inhibitor as control group. In RAS inhibitor group, 57 subjects had angiotensin II receptor blocker, 2 subjects had angiotensin-converting-enzyme inhibitor, and 1 subject had renin inhibitor. The study protocol was approved by our hospital ethics committee (no. 2419).

Clinical characteristics at baseline in RAS inhibitor group (*n* = 60, male/female = 35/25) and control group (*n* = 100, male/female = 60/40) were as follows: age, 60.0 ± 1.1 vs. 63.0 ± 1.4 years old (not significant (n.s.)); duration of diabetes, 11.3 ± 1.1 vs. 9.5 ± 1.1 years (n.s.); HbA1c, 7.09 ± 0.09% vs. 7.01 ± 0.13% (n.s.); Hb, 14.0 ± 0.2 g/dL vs. 13.6 ± 0.1 g/dL (n.s.); BMI, 23.8 ± 0.4 kg/m^2^ vs. 26.3 ± 0.8 kg/m^2^ (*p* < 0.05); LDL cholesterol, 103.2 ± 2.7 mg/dL vs. 104.4 ± 3.5 mg/dL (n.s.); HDL cholesterol, 54.0 ± 1.6 mg/dL vs. 55.1 ± 2.5 mg/dL (n.s.); triglyceride, 131.9 ± 9.3 mg/dL vs. 127.6 ± 10.8 mg/dL (n.s.); systolic blood pressure, 122.1 ± 1.8 mmHg vs. 130.3 ± 2.0 mmHg (*p* < 0.05); diastolic blood pressure, 70.6 ± 1.3 mmHg vs. 72.8 ± 1.6 mmHg (n.s.); urinary albumin excretion (UAE), 13.3 ± 0.6 mg/gCr vs. 13.6 ± 0.9 mg/gCr (n.s.); eGFR, 83.8 ± 2.3 mL/min/1.73m^2^ vs. 79.4 ± 2.3 mL/min/1.73m^2^; uric acid, 5.6 ± 0.2 mg/dL vs. 5.3 ± 0.1 mg/dL (n.s.); prevalence of diabetic retinopathy, 14.3% vs. 9.3% (n.s.); smoking status, 40.8% vs. 44.4% (n.s.); and family history of diabetes, 55.6% vs. 58.1% (n.s.). There was no difference in the ratio of antidiabetic drug use between the 2 groups. Similarly, there was no difference in the ratio of lipid-lowering drug use between the 2 groups: statin, 45.0% vs. 46.0% (RAS inhibitor group vs. control group) (n.s); fibrates, 8.3% vs. 6.0% (n.s.); and ezetimibe, 55.6% vs. 58.1% (n.s.).

An analysis of the relative odds of the occurrence of the outcome was performed with the use of a logistic regression model.

## 3. Results

**Figure 1:**
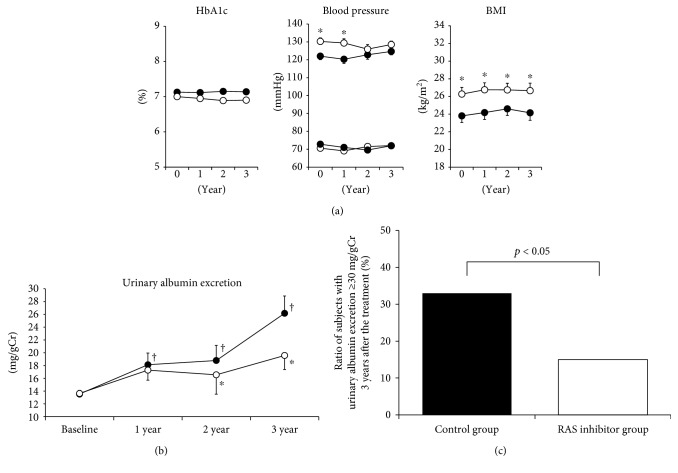
(a) Alteration of HbA1c, blood pressure, and BMI during 3-year observational period. Closed circle, control group; open circle, RAS inhibitor group. ^∗^*p* < 0.05. (b) Alteration of urinary albumin excretion during 3-year observational period. Closed circle, control group; open circle, RAS inhibitor group. ^∗^*p* < 0.05 vs. control group, ^†^*p* < 0.05 vs. baseline value in control group. (c) Ratio of subjects with urinary albumin excretion ≥ 30 mg/gCr in subjects with and without RAS inhibitor treatment.

There was no difference in HbA1c levels between RAS inhibitor group and control group through the 3-year observation period (Figure 1(a)). Systolic blood pressure in RAS inhibitor group was higher compared to that in control group at baseline and 1 year after starting RAS inhibitor (*p* < 0.05), but there was no difference between them 2 and 3 years after the treatment. There was no difference in diastolic blood pressure between the 2 groups. BMI in RAS inhibitor group was higher compared to that in control group through the observation period (*p* < 0.05).

Urinary albumin excretion (UAE) in control group was significantly increased compared to baseline (from 13.5 ± 0.5 mg/gCr to 26.2 ± 2.2 mg/gCr) (*p* < 0.05), whereas there was no significant difference in RAS inhibitor group between at baseline and 3 years after the treatment (13.6 ± 0.6 mg/gCr and 19.6 ± 2.7 mg/gCr) (Figure 1(b)). The alteration of UAE in RAS inhibitor group and control group was 2.5 ± 1.6 mg/gCr and 11.4 ± 2.2 mg/gCr, respectively. In addition, 2 and 3 years after the treatment, there was significant difference in UAE between the 2 groups (*p* < 0.05). UAE in 33% of control group increased up to ≥30 mg/gCr whereas only 15% of RAS inhibitor group increased up to ≥30 mg/gCr (Figure 1(c)). These data suggest that RAS inhibitor therapy exerts protective effect on the progression of UAE.

**Figure 2:**
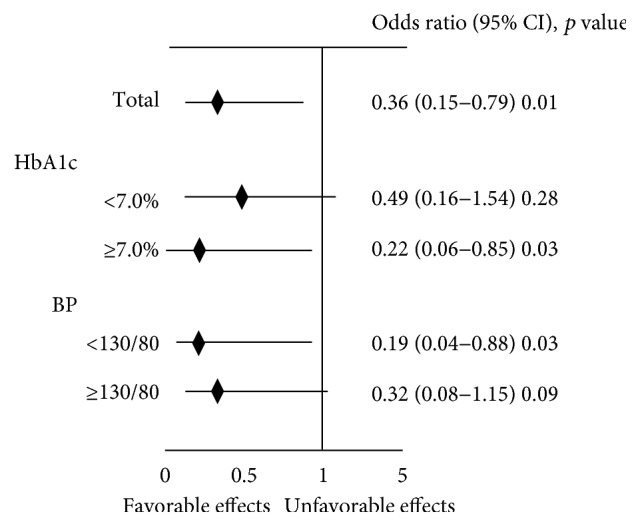
Odds ratio (95% CI) for favorable effect of RAS inhibitor on urinary albumin excretion. Comparison of odds ratio between the subjects with HbA1c < 7.0% and ≥7.0% and between the subjects with blood pressure < 130/80 mmHg and ≥130/80 mmHg.

Next, we evaluated odds ratio about the progression of diabetic nephropathy. As shown in Figure 2, odds ratio (95% CI) for favorable effect of RAS inhibitor on UAE in all subjects was 0.36 (0.15–0.79), suggesting that RAS inhibitor exerted favorable effects. Odds ratio for favorable effect of RAS inhibitor on UAE in subjects with HbA1c < 7.0% and ≥7.0% was 0.49 (0.16–1.54) and 0.22 (0.06–0.85), respectively. These data suggest that RAS inhibitor exerted more beneficial effects in subjects with poor glycemic control. Odds ratio for favorable effect of RAS inhibitor on UAE in subjects with blood pressure < 130/80 mmHg and ≥130/80 mmHg was 0.19 (0.04–0.88) and 0.32 (0.08–1.15), respectively. These data suggest that blood pressure control is important for RAS inhibitor to exert beneficial effect.

Finally, to evaluate the possible association between the alteration of UAE and various clinical parameters, we performed univariate analysis. As shown in Table 1, the alteration of UAE was closely associated with annual mean HbA1c level through 3-year observational period in control group (*r* = 0.406, *p* < 0.0005), but such association was not observed in RAS inhibitor group (*r* = 0.003, *p*: n.s.). There was no association in both groups between the alteration of UAE and various other clinical parameters including age, duration of diabetes, eGFR at baseline, and annual mean of HbA1c levels, systolic and diastolic blood pressure, CRP, BMI, triglyceride, and LDL and HDL cholesterol.

Furthermore, to adjust the possible influence of blood pressure, eGFR, age, and gender on the alteration of UAE, we performed multivariate analysis using annual mean of HbA1c levels, systolic blood pressure, GFR and age at baseline, and gender as explanatory variables and the alteration of UAE as an objective variable. As shown in Table 2, annual mean of HbA1c levels was an independent determinant factor for the alteration of UAE in control group (*β* = 7.574, *p* < 0.005), but not in RAS inhibitor group (*β* = −1.749, *p*: n.s.). These data strengthened the idea that RAS inhibitor could protect renal function against the deleterious effect of chronic hyperglycemia in subjects with type 2 diabetes without diabetic nephropathy.

## 4. Discussion

It was known that RAS inhibitors suppressed the progression of overt diabetic nephropathy, but it remained unclear whether RAS inhibitors would bring out some favorable effects on renal function even before the onset of diabetic nephropathy. In this study, we showed that RAS inhibitor could exert protective effect on the progression of UAE and could counteract the adverse effect of hyperglycemia in subjects with type 2 diabetes even before the onset of diabetic nephropathy (Figure 1). RAS inhibitor exerted more protective effects on renal function in subjects with poor glycemic control (Figure 2). Therefore, it is likely that administration of RAS inhibitor reduces the adverse effect of hyperglycemia on the progression of UAE. In addition, these data suggest that it is more important to use RAS inhibitor when glycemic control is poor. Poor glycemic control was an independent determinant factor for the alteration of UAE in control group, but such findings were not observed in RAS inhibitor group (Table 2). These data suggest that deleterious effect of chronic hyperglycemia on renal function was reduced by the usage of RAS inhibitor in subjects with type 2 diabetes before the onset of diabetic nephropathy. In addition, RAS inhibitor exerted more protective effects on renal function in subjects with good control of blood pressure (Figure 2). These data suggest that it is very important to maintain good control of blood pressure so that RAS inhibitor could bring out more favorable effect on the progression of UAE and protection of renal function. Taken together, it is likely that RAS inhibitor exerts more beneficial effects in subjects with poor glycemic control and good control of blood pressure.

There are several limitations in this study. First, this is a retrospective study, but not a prospective study. Therefore, it would be necessary to perform a prospective study with a placebo control in order to strengthen our hypothesis. Second, the data in this study are influenced by lifestyle, diet, and other factors such as alternative medicine. Therefore, it would be necessary to perform some intervention to various factors including lifestyle so that we can demonstrate our hypothesis.

In conclusion, RAS inhibitor could protect renal function against the deleterious effect of chronic hyperglycemia in Japanese subjects with type 2 diabetes even before the onset of diabetic nephropathy.

## Figures and Tables

**Table 1:** Association between the alteration of urinary albumin excretion and various clinical parameters: univariate analysis.

	Control group		RAS inhibitor group	
	*r*	*p*	*r*	*p*
HbA1c, annual mean	0.406	<0.0005	0.003	n.s.
Age, baseline	−0.083	n.s.	0.146	n.s.
Duration of diabetes, baseline	−0.002	n.s.	−0.104	n.s.
Systolic BP, annual mean	0.021	n.s.	0.250	n.s.
Diastolic BP, annual mean	0.041	n.s.	−0.004	n.s.
CRP, annual mean	0.199	n.s.	−0.090	n.s.
TG, annual mean	0.093	n.s.	0.069	n.s.
HDL-C, annual mean	−0.146	n.s.	−0.083	n.s.
LDL-C, annual mean	−0.031	n.s.	0.101	n.s.
BMI annual mean	0.035	n.s.	−0.033	n.s.
eGFR, baseline	0.202	n.s.	0.066	n.s.

Abbreviations: n.s., not significant; BP, blood pressure; TG, triglyceride; LDL-C, low-density lipoprotein cholesterol; HDL-C, high-density lipoprotein cholesterol; BMI, body mass index; RAS, renin-angiotensin system.

**Table 2:** Association between the alteration of urinary albumin excretion and various clinical parameters: multivariate analysis.

	Control group		RAS inhibitor group	
	*β*	*p*	*β*	*p*
HbA1c, annual mean	7.574	<0.005	−1.749	n.s.
Age, baseline	0.208	n.s.	0.171	n.s.
Systolic BP, annual mean	−0.021	n.s.	0.165	n.s.
Gender	0.533	n.s.	1.017	n.s.
eGFR, baseline	0.019	n.s.	0.142	n.s.

Abbreviations: n.s., not significant; BP, blood pressure; RAS, renin-angiotensin system.

## Data Availability

The data used to support the findings of this study are available from the corresponding author upon request.
